# Adult Cardiac Expression of the Activating Transcription Factor 3, ATF3, Promotes Ventricular Hypertrophy

**DOI:** 10.1371/journal.pone.0068396

**Published:** 2013-07-03

**Authors:** Lilach Koren, Ofer Elhanani, Izhak Kehat, Tsonwin Hai, Ami Aronheim

**Affiliations:** 1 Department of Molecular Genetics, the Rappaport Family Institute for Research in the Medical Sciences, Technion-Israel Institute of Technology, Haifa, Israel; 2 Department of Physiology The Rappaport Family Institute for Research in the Medical Sciences, Technion-Israel Institute of Technology, Haifa, Israel; 3 Department of Molecular and Cellular Biochemistry, Ohio State University, Columbus, Ohio, United States of America; Virginia Commonwealth University, United States of America

## Abstract

Cardiac hypertrophy is an adaptive response to various mechanophysical and pathophysiological stresses. However, when chronic stress is sustained, the beneficial response turns into a maladaptive process that eventually leads to heart failure. Although major advances in the treatment of patients have reduced mortality, there is a dire need for novel treatments for cardiac hypertrophy. Accordingly, considerable efforts are being directed towards developing mice models and understanding the processes that lead to cardiac hypertrophy. A case in point is ATF3, an immediate early transcription factor whose expression is induced in various cardiac stress models but has been reported to have conflicting functional significance in hypertrophy. To address this issue, we generated a transgenic mouse line with tetracycline-regulated ATF3 cardiac expression. These mice allowed us to study the consequence of ATF3 expression in the embryo or during the adult period, thus distinguishing the effect of ATF3 on development versus pathogenesis of cardiac dysfunction. Importantly, ATF3 expression in adult mice resulted in rapid ventricles hypertrophy, heart dysfunction, and fibrosis. When combined with a phenylephrine-infusion pressure overload model, the ATF3 expressing mice displayed a severe outcome and heart dysfunction. In a complementary approach, ATF3 KO mice displayed a lower level of heart hypertrophy in the same pressure overload model. In summary, ectopic expression of ATF3 is sufficient to promote cardiac hypertrophy and exacerbates the deleterious effect of chronic pressure overload; conversely, ATF3 deletion protects the heart. Therefore, ATF3 may serve as an important drug target to reduce the detrimental consequences of heart hypertrophy.

## Introduction

Heart failure affects approximately 1–3% of the population in the developed world. The incidence of heart failure increases with age affecting 10 percent of the population over the age of 70 [1]. The development of heart failure is associated with cardiac hypertrophy and remodeling [2]. Hypertrophy is a hallmark of cardiac remodeling in which the heart exhibits an increase in size without any significant cardiomyocytes proliferation. The cardiomyocytes in a hypertrophic heart show phenotypic modifications which include the re-expression of the fetal gene program, abnormal Ca^+2^ handling, oxidative stress, mitochondrial damage, collagen deposition, and metabolic changes. The altered gene expression program is the result of changes in the activity levels of key regulatory transcription factors that mediate the hypertrophic gene expression signature and outcome.

ATF3 is a member of the basic leucine zipper (bZIP) family of transcription factors. The leucine zipper domain mediates the dimerization with various members of the bZIP family and the basic domain is responsible for binding to specific DNA sequences, which are known collectively as ATF/AP-1 elements. Depending on its dimerization partner, target promoter, and cellular context, ATF3 can act either as a transcriptional activator or repressor [3,4]. ATF3 potentiates transcription following hetero-dimerization with Chop10 [5] or c-Jun [3]. Alternatively, ATF3 represses transcription as a homodimer by recruiting multiple members of the histone deacetylase proteins (HDACs) to target gene promoters [6]. ATF3 is encoded by an immediate early gene that is highly induced in response to multiple cell stresses [7,8]. The baseline ATF3 mRNA level is low, but greatly increases following pleiotropic stimuli. ATF3 plays a central role in the rapid regulation of a large number of target genes and is considered a hub for the cellular adaptive response to signals that perturb homeostasis [8]. In the heart, many insults and signals have been shown to induce ATF3, including ischemia/reperfusion [9], doxorubicin [10], and neurohormonal signals such as the α and β adrenergic agonists isoprterenol and phenylephrine and angiotensin II [11].

The functional importance of ATF3 in cardiac hypertrophy is somewhat controversial. ATF3 deficiency (a loss-of-function approach) has been shown to promote cardiac hypertrophy in an aortic banding pressure overload model [12]. In addition, an ATF3 knock down experiment resulted in an improper response to endothelin-induced cardiomyocyte hypertrophy in an *in vitro* model [13], suggesting a beneficial effect of ATF3. Conversely, transgenic mice with cardiac ATF3 expression (a gain-of-function approach) resulted in enlarged atria, fibrosis, conduction defects, and sudden death [9], suggesting a deleterious effect of ATF3. One explanation for this apparent discrepancy is the difference in the approach, loss-versus gain-of-function. It is important to note that in the transgenic model, ATF3 expression was under the control of the αMHC promoter, which is turned on in the atria at embryonic day 10 and in the ventricles shortly before birth [14]. Therefore, it is difficult to conclude whether the phenotype is due to the developmental effect of expressing ATF3 in the embryos or a bona fide functional consequence of ATF3 expression in the adult heart. To differentiate between these two possibilities, we generated transgenic mice with regulated expression of ATF3 using the tetracycline-inducible system. We also investigated the role of ATF3 in cardiac hypertrophy induced by pressure overload using these transgenic mice. To complement this gain-of-function approach, we used knockout (KO) mice deficient in ATF3 and compared their phenotypes under pressure overload to that of the wild type mice. Collectively, our data are consistent with the model that ATF3 promotes cardiac hypertrophy, both alone and together with chronic pressure overload.

## Materials and Methods

### Transgenic Design

The hemagglutinin (HA) epitope-tagged ATF3 cDNA was inserted into the bi-cistronic pBI-G expression plasmid (Clontech Inc.). The tet-promoter is designed to bi-directionally drive the β-galactosidase gene and HA-ATF3. Linearized plasmid was injected into the oocytes and several germ line transmitting mouse lines with a variable number of ATF3 integration copies. One of these lines was crossed with an α-MHC-tTA driver line (kindly provided by Prof. E. Keshet) to direct the expression in the heart [15].

Genotyping was performed on genomic mouse tail DNA that was extracted using the Redextract-N-AMP tissue PCR kit (Sigma, St. Louis, MO, USA).

ATF3 forward primer ATGGGATCCACCATGTACGACG
ATF3 reverse primer CCGGAATTCGGGCTCTGCAATG
Tet forward primer GCTGCTTAATGAGGTCGGAATCG
Tet reverse primer GCCCCACAGCGCTGAGTGCAT

### Chemicals

Phenylephrine (Sigma P6126); Doxycyline hyclate (Sigma D9891) was diluted in drinking water containing 5% sucrose to final concentration of 0.2 mg/ml.

### Mice

This study was carried out in strict accordance with the Guide for the Care and Use of Laboratory Animals of the National Institutes of Health. The protocol was approved by the Committee on the Ethics of Animal Experiments of the Technion (Permit Number: IL-029-03-2009, IL-035-03-2011). Surgery was performed under sodium pentobarbital anesthesia, and all efforts were made to minimize suffering. The animals were fed standard rat chow containing 0.5% NaCl and tap water ad libitum. The ATF3 transgenic mice were under FVB background and ATF3 KO mice represent C57Bl/6 background [9].

### Mice Injections

C57Bl/6 mice were injected intraperitoneally with 2.5 mg/kg of phenylephrine. At the indicated time following injection, the mice were anesthetized using ketamine and xylazine and heart chambers were separated until further extraction.

### Micro-Osmotic Pumps Implantation

Alzet micro-osmotic pumps (#1002, Alzet) were filled with either Phenylephrine (100 mg/Kg/day, 0.06% acetic acid in saline) or saline. Mice were anesthetized with sodium pentobarbital and were subcutaneously implanted with pumps. The procedure was performed under sterile conditions. The mice were weighed and sacrificed 14 days after this procedure. The ventricles’ weights were determined after separation from the atria (referred to hereafter as “ventricles weight”). The ventricles were then divided into three pieces that were used for protein extraction, RNA purification, and tissue fixation in 4% formaldehyde overnight, respectively. Atria were either fixed in 4% formaldehyde or used for protein or RNA extraction as well.

### Histology

Heart tissues were fixed in 4% formaldehyde for at least overnight, then embedded in paraffin, serially sectioned at 10 µm intervals, and mounted on slides. Sections were processed for deparaffinization (xylene, 20 min), dehydration (isopropanol), rehydration (H_2_O), and antigen unmasking (10 mM sodium citrate pH-6, 90^0^C, 12 min). Immunostaining was performed using Histostain kit (#956143, Invitrogen) according to the manufacturer’s instructions. Anti-ATF3 antibodies were diluted 1:100. Following immunostaining, nuclei were stained with hematoxylin. Masson’s trichrome staining was performed according to standard protocol.

### Cell Size Analysis and Quantification

Sections were stained following deparaffinization with wheat-germ agglutinin TRITC-conjugated (Sigma Aldrich Cat# L5266) diluted 1:100 in phosphate-buffered saline (PBS). Sections were washed three times with PBS and mounted in Fluoromount-G (Southern Biotechnology, Birmingham, AL, 0100-01). Sections were viewed using a Zeiss LSM 510 meta-inverted confocal microscope (Thornwood, NY) equipped with a 40X oil objective with a DPSS laser (561 nm).

Quantification of the cell size was performed with Image Pro Plus software. Five fields in each slide were photographed. Unstained areas were then identified and segmented using Image Pro Plus software. In each stained area, the mean cell perimeter and area was calculated by an experimentalist blinded to the experimental groups.

### mRNA Extraction

mRNA was purified using an Aurum total RNA fatty and fibrous tissue kit (#732-6830, Bio-Rad) according to the manufacturer’s protocol. mRNA was quantified by measuring Ab260 nm with a nanodrop spectrophotometer (ND- 1000, NanoDrop Technologies, Rockland, DE ,USA).

### Quantitative Real Time PCR (RT-qPCR)

cDNA was synthesized from mRNA samples using 800 ng of RNA in a 20µl total reaction mix of high-capacity cDNA reverse transcription kit (#4368814, Applied Biosystems). Real-time PCR was performed using Rotor-Gene 6000TM (Corbett) equipment with absolute blue SYBER green ROX mix (Thermo Scientific AB-4162/B). Serial dilutions of a standard sample were included for each gene to generate a standard curve. Values were normalized with β2 microglobulin or GAPDH expression levels. The primer sequences are shown in Table 1. Additional quantifications were performed by RT-qPCR Taqman gene expression assay (Applied Biosystems Inc.) according to the manufacturer’s recommended protocol. The primers used were: Myh7 (β-MHC) #Mm08600555_m1, Nppa (ANP) #Mm01255748_g1, and b2m (β2 microtubulin) # Mm00437762_m1. Calculations for Taqman-analyzed transcripts were performed using the delta-delta Ct method.

**Table 1:** RT-qPCR mouse primer sequences used.

Primer name	Sequence 5'–3'
mATF3	F-GCTGCTGCCAAGTGTCGAAA R-TACATGCTCAACCTGCACCG
hATF3	F-AAGAACGAGAAGCAGCATTTGAT R-TTCTGAGCCCGGACAATACAC
GAPDH	F-TTGCCATCAACGACCCCTTCAT R-AGACTCCACGACATACTCAGCA
Acta1	F-GTGAGATTGTGCGCGACATC R-GGCAACGGAAACGCTCATT
Col1α	F-CTGGCGGTTCAGGTCCAAT R-TTCCAGGCAATCCACGAGC
cTGF	F-AGACCTGTGGGATGGGCAT R-GCTTGGCGATTTTAGGTGTCC
TGFβ	F-CCTGGCCCTGCTGAACTTG R-GACGTGGGTCATCACCGAT
MLC2a	F-GGCACAACGTGGCTCTTCTAA R-TGCAGATGATCCCATCCCTGT
MLC2v	F-ATCGACAAGAATGACCTAAGGGA R-ATTTTTCACGTTCACTCGTCCT
IL-6	F-TAGTCCTTCCTACCCCAATTTCC R-TTGGTCCTTAGCCACTCCTTC
CD68	F-TGTCTGATCTTGCTAGGACCG R-GAGAGTAACGGCCTTTTTGTGA
F4/80	F-CCCCAGTGTCCTTACAGAGTG R-GTGCCCAGAGTGGATGTCT
BNP	F-GAGGTCACTCCTATCCTCTGG R-GCCATTTCCTCCGACTTTTCTC

### Echocardiography

Mice were anesthetized with 1% isoflurane and kept at 37^o^C. The echocardiography was performed using a Vevo2100 Micro-ultrasound imaging system (Visualsonics) equipped with 13-38MHz (MS 400) and 22-55MHz (MS550D) array transducers. Conventional two-dimensional imaging and M-Mode recordings were performed to determine cardiac size, shape, and function. Measurements were recorded to determine the fractional shortening (FS) percentage to assess heart function. Maximal left ventricles end-diastolic (LVDd) and end-systolic (LVDs) dimensions parameters were measured in short-axis M-mode images. Fractional shortening (FS) was calculated as FS (%) = [(LVDd-LVDs)/LVDd] X 100. All values were based on the average of at least three measurements.

### Cell Culture and Transient Transfection

Human embryonic kidney 293T cells (HEK-293T) were maintained in DMEM containing 10% FCS and 1% penicillin and streptomycin and grown at 37°C and 5% CO_2_. HEK-293T cells were co-transfected with the appropriate expression plasmids using the calcium-phosphate (Ca_2_PO_4_) method. The total amount of plasmid DNA was adjusted to 10–12 µg in a total volume of 1000 µl. Cells were replaced with fresh medium 4–5 h after transfection and harvested 24 h after transfection.

### Western Blotting

Cells were lysed in whole-cell extract (WCE) buffer (25 mM HEPES, pH 7.7, 0.3 M NaCl, 1.5 mM MgCl_2_, 0.2 mM EDTA, 0.1% Triton X-100, 0.5 mM DTT, 20 mM β-glycerolphosphate, 0.1 mM Na _2_VO_4_, 100 µg/ml PMSF, protease inhibitor cocktail 1:100; Sigma-Aldrich, P8340).

Harvested tissues were homogenized in RIPA buffer (1% NP-40, 5 mg/ml Na-deoxycholate, 0.1% SDS in PBSX1) supplemented with protease inhibitors cocktail-P-8340, Sigma Aldrich; 1 mM DTT ; 2 mM PMSF ; 20 mM β-glycerolphosphate ; 0.1 mM Sodium vanadate; 20 mM PNPP ; PhosStop- #04906837001, Roche. Homogenization was performed at 4^0^C using the Bullet Blender homogenizer (BBX24; Next advance) according to manufacturer’s instructions. Next, lysates were centrifuged at maximal speed for 10 minutes and supernatants were frozen at -70^0^C.

The proteins (30 µg of cell lysate or 80 µg of tissue lysate) were then separated by 12.5% SDS-PAGE, followed by a transfer to a nitrocellulose membrane. Blots were blocked in 5% dry milk in PBS and washed three times for 5 min in PBS. The primary antibodies used were anti-α-tubulin (T-9026, Sigma Aldrich) 1:2000, anti-HA 1:500, and anti-ATF3 1:100. Primary antibodies were incubated for at least 1 h at 4°C. Primary antibodies were detected using the corresponding HRP-conjugated secondary antibodies obtained from Sigma-Aldrich.

### Statistical analysis

Data is presented as means ± SEM in (n) number of experiments.

Differences were analyzed using one-tailed Student’s t-test (unless otherwise indicated), with an assumption of equal variance.

The Mendelian ratio calculation was analyzed by a χ^2^ test for expected-versus-observed ratio*.*

P values < 0.05 were considered significant unless otherwise indicated.

## Results

**Figure 1:**
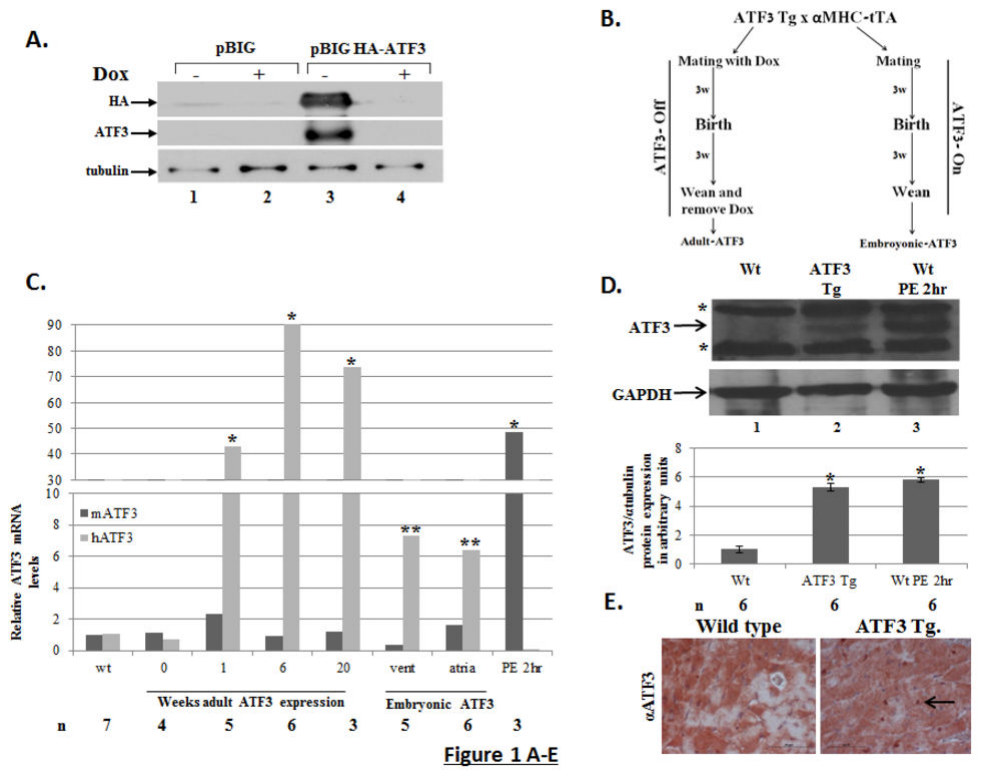
HA-ATF3 transient expression is tightly controlled by doxycycline. **A**. pBI-G expression vector plasmid or HA-ATF3 expression plasmid (pBIG-HA-ATF3) were co-transfected with the tTA (tet-off) expression plasmid into HEK-293T cells in the presence (+) or absence (-) of doxycycline (Dox, 10µg/ml). Nuclear cell lysate was separated on 12.5% SDS-PAGE followed by Western blot analysis with anti-HA, anti-ATF3, and anti-α tubulin (loading control). **B**. Schematic representation of the mating cages. Gender matched heterozygotes αMHC-tTA driver mice were mated with ATF3-tg responder mice line. Mating in the presence of doxycycline represents ATF3-Off expression during embryonic development therefore, mice are designated adult-ATF3 expressing. Mating in the absence of doxycycline represents ATF3-On expression through embryonic development therefore, mice are designated embryonic-ATF3 expressing. **C**. RT-qPCR analysis for cDNA derived from atria and ventricles of either wild-type or ATF3 transgenic mice treated with doxycycline as indicated. The expression level of ATF3 was examined by either mouse- (black) or human-specific primers (gray). The results represent the mean expression relative to GAPDH of the indicated number of animals (n). Asterisks (*/**) represent P value <0.05 or <0.01 of a one-tailed t-test compared to wild-type mice. **D**. Representative Western blot analysis (Top panel) of cell lysates derived from ventricles of wild type mice (Wt.), adult-ATF3 expressing and wild type mice following 2 h PE injection. The membrane was probed with anti-ATF3 and GAPDH for loading control. The asterisks (*) represent non-specific cross reactive proteins (non-specific). Densitometry analysis (bottom panel) of ATF3 expression was normalized with the GAPDH level. The results represent the mean and SEM from six independent animals. **E**. Immunohistochemistry of left ventricle sections stained with αATF3 (1:200). Representative sections derived from mice positive for HA-ATF3 responder and αMHC driver (ATF3 Tg, right panel) and Wild-type mouse (left panel). The magnification shown is X20. The black arrow indicates an ATF3-stained nucleus.

We initially used a gain-of-function approach to test whether ATF3 is sufficient to induce ventricles hypertrophy in the adult heart using transgenic mice expressing ATF3 by the tet-off system. We generated a pBI-G derivative plasmid expressing a hemagglutinin-tagged human ATF3 (pBIG-HA-ATF3) under the control of the tetracycline response elements. HEK-293T cells were transfected with pBIG-HA-ATF3 expression plasmid together with pRetro-tTA (tet-off) plasmid, which expresses the tet-off transcriptional activator. Following transfection, cells were either treated with doxycycline (Dox +) or left untreated (Dox -) and the HA-ATF3 expression level was examined by Western blot analysis (Figure 1A). ATF3 was readily detected in the lysate derived from cells grown in the absence of doxycycline. In contrast, no expression was observed in the cells grown in the presence of doxycycline (Figure 1A). We then generated ATF3 responder transgenic mice using pBIG-HA-ATF3 by pronuclear injection and crossed the responder mice with the αMHC-tTA driver mice [15]. We set two groups of mating cages in which mice were either provided with doxycycline all the time till weaning to express ATF3 at weaning (referred to as adult-ATF3 expressing) or never treated with doxycycline, allowing HA-ATF3 expression as soon as αMHC-tTA was turned on in embryos (referred to as embryonic-ATF3 expressing) (Figure 1B).

The expression of human ATF3 transgene was evaluated using reverse transcription coupled with quantitative real-time PCR (RT-qPCR) with appropriate primers that discriminate between the transgenic human ATF3 and the endogenous mouse ATF3 transcripts (Figure 1C). Double-transgenic mice fed with doxycycline displayed very low levels of transgene expression (Figure 1C, 0 weeks) demonstrating tight regulation of the transgene expression in the presence of doxycycline. In contrast, the double-transgenic mice that had doxycycline removed upon weaning (adult-ATF3 expressing mice) displayed relatively high levels of human ATF3 expression within a week, which remained high through adulthood (Figure 1C, light gray, 1, 6 and 20 weeks). The induction of transgenic ATF3 at mRNA level (~40-90 fold relative to the endogenous un-induced level of ATF3 mRNA) is comparable to (albeit slightly higher than) that of the endogenous ATF3 following two hours after the phenylephrine injection (~50 fold) (Figure 1C, PE 2hr). The ATF3 protein level of the transgene is about the same level as the endogenous ATF3 protein following PE acute injection (Figure 1D). Therefore, the transgene represents a chronic ATF3 expression of a relatively physiological relevant expression level. Immunohistochemistry analysis of ventricular sections displayed nuclear ATF3 staining in the adult-ATF3 expressing heart (Figure 1E, see discussion for the low number of cells positive for ATF3).

The driver and responder were maintained as heterozygous for either the tTA or the ATF3 transgene. We expected to obtain double-transgenic mice at a frequency rate of 25%. Whereas the offspring derived from doxycycline-treated mice displayed the expected Mendelian ratio (23.9-27.8%, from n=247) (Figure S1A), the mating cages in which doxycycline was avoided (which allowed ATF3 expression during embryonic development) displayed a significantly lower percentage of double-positive mice (16.1% from n=118) (Figure S1A). This analysis suggests that some of the ATF3 expressing embryos died before birth. In addition, only 50% of the constitutive-ATF3 expressing mice survived beyond eight weeks of age (Figure S1B). These mice displayed a low level of ATF3 compared to the adult-ATF3 expressing mice (Figure 1B). Importantly, the embryonic-ATF3 expressing mice sacrificed prior to death showed significant atria enlargement (Figure S1C). Their atria-to-body weight ratio was 6–7 times higher than that of wild-type mice (Figure S1D). Although their ventricle-to-body weight ratio was slightly higher than that of the wild type mice, the difference is not statistically significant. The embryonic-ATF3 expressing mice were examined prior to death by micro-ultrasound and electrocardiography (ECG). ECG recordings showed that the mice suffered from arrhythmias (Figure S1E), and probably died from an AV-block.

Hearts derived from embryonic-ATF3 expressing mice displayed elevated levels of early embryonic marker brain natriuretic peptide (BNP, Figure S2A). Staining by TRITC-labeled wheat-germ agglutinin (to demarcate the cell boundary) showed an increase in cell size in the transgenic atria compared to wild-type non-transgenic counterpart (Figure S2 B-C). Analysis of ventricles size showed a similar trend (larger transgenic than non-transgenic cell size), but the difference was not statistically significant.

Furthermore, atria derived from embryonic-ATF3 expressing mice displayed higher levels of fibrotic markers, such as transforming growth factor β (TGFβ), collagen type1α (col1α), and connective tissue growth factor (cTGF) (Figure S2 D–F). Interestingly, the corresponding ventricles that displayed only minor morphologic changes expressed elevated levels of TGFβ and cTGF (Figures S2D and S2F).

An earlier report of another bZIP repressor protein, JDP2 [16], suggested that the enlarged phenotype observed can be explained by the suppression of MLC2a and connexin 40 (CX40) gene expression in JDP2 transgenic mice [17–20]. To examine whether ATF3 expression resulted in down-regulation of MLC2a and CX40 expression, we performed RT-qPCR using mRNA derived from atria of either wild-type or embroyonic-ATF3 transgenic mice. Indeed, we observed a five-fold reduction in MLC2a and a ten-fold reduction of CX40 transcripts in the embryonic-ATF3 expressing transgenic mice compared with wild-type mice (Figure S3A-B).

To assess heart function at weaning time (three weeks of age) in these embryonic-ATF3 expressing mice, we determined heart function by calculating the fractional shortening (FS) percentage. The average calculated FS in embryonic-ATF3 expressing mice was 24% ± 3.7, compared to 43%± 4.2 in the wild type non-transgenic mice.

Collectively, mice expressing ATF3 during embryonic development acquired enlarged atria, arrhythmia, and early death. These data are consistent with the previous report that showed similar phenotypes using transgenic mice expressing ATF3 in the embryos by the αMHC promoter [9].

**Figure 2:**
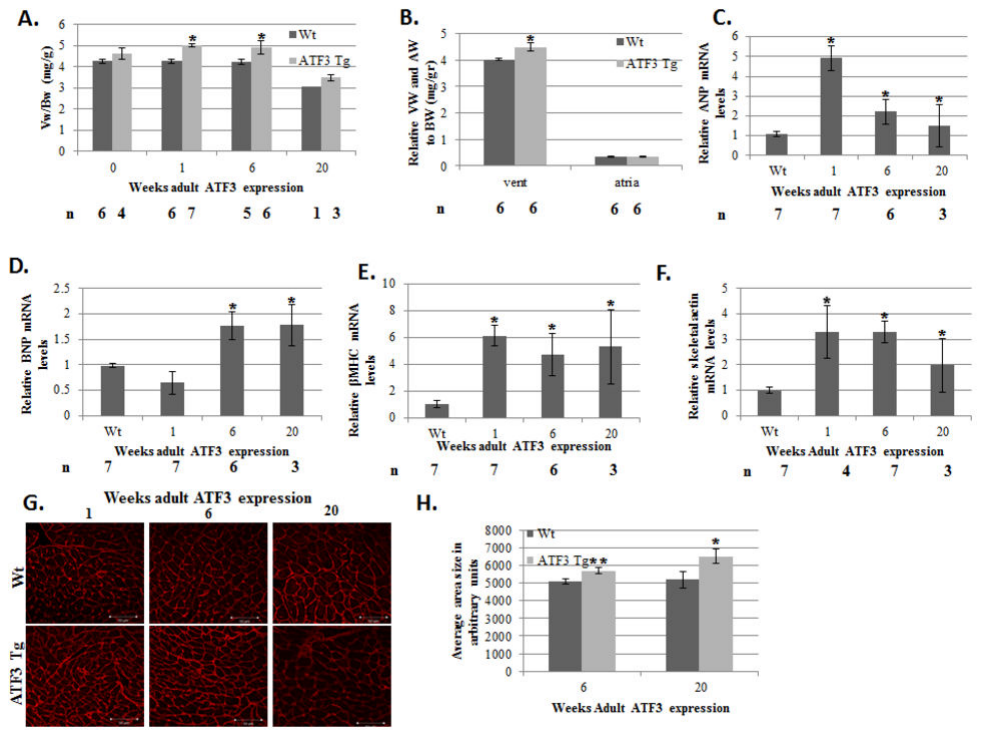
Adult-ATF3 expression promotes hypertrophy. A. Mice were mated in the presence of doxycycline (adult ATF3 expressing). Weaned newborn mice were either maintained with doxycycline containing water (0 weeks without doxycycline) or provided with regular water. Mice were sacrificed at the indicated number of weeks following doxycycline removal. Mice ventricles were weighed (Vw) to mouse body weight (Bw). The results represent the ratio of Vw/Bw (mg/gr) of ATF3 transgenic mice (gray) and wild-type (black) mice at the indicated time (weeks) following doxycycline removal. The results represent the mean and SEM of the indicated number of animals (n). **B**. Atria and ventricles weight relative to body weight at 6 weeks of age (mg/gr). **C**–**F**. Adult-ATF3 expressing mice show higher expression of hypertrophic markers. RT-qPCR analysis for cDNA derived from RNA extracted from ventricles of ATF3-transgenic and wild-type mice with the corresponding specific primers to the following genes: **C**. Atrial natriuretic peptide (ANP) D. Brain natriuretic peptide (BNP) **E**. β Myosin heavy chain (βMHC) F. Skeletal actin (Acta1). The results represent the mean expression relative to GAPDH of the indicated number of animals (n). **G**. Ventricles sections were stained with TRITC-labeled wheat germ aglutinin and the cell size was analyzed using the Image Pro Plus software. **H**. Quantification of cell size in G. The results represent the mean and SEM from five different sections derived from wild type (n=2) and adult ATF3 expressing (n=3) animals at the indicated time following doxycycline removal. Asterisks (*/**) represent P value <0.05 or <0.01 respectively of a one-tailed t-test compared with wild-type mice.

**Figure 3:**
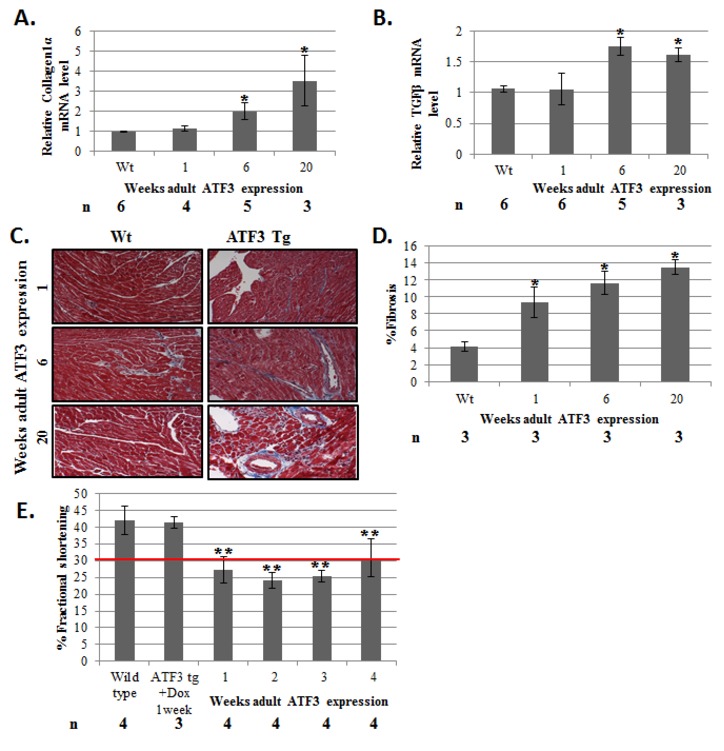
The hearts derived from adult-ATF3 expressing mice display a higher level of fibrosis and lower heart function. mRNA described in Figure 2C was analyzed for the indicated fibrosis markers A. Collagen 1α (Col1α) **B**. Transforming growth factor β (TGFβ). The results represent the mean expression relative to GAPDH of the indicated number of animals (n). **C**. Representative Masson Trichrome staining of paraffin embedded sections of wild-type and adult-ATF3 expressing mice at the indicated time following doxycycline removal **D**. Quantification of fibrosis of the indicated number of mice (n). At least five sections per mice were analyzed. **E**. Adult-ATF3 expressing mice treated as indicated were examined by micro-ultrasound and measurements were recorded to determine the fractional shortening (FS) percentage. Maximal left ventricles end-diastolic (LVDd) and end-systolic (LVDs) dimensions parameters were measured in short-axis M-mode images. Fractional shortening (FS) was calculated as: FS (%) = [(LVDd-LVDs)/LVDd] X 100. Echocradiography measurements were performed at the indicated number of weeks following doxycycline removal. The results represent the mean and SEM of the indicated number of animals (n). Asterisks (*/**) represent P value <0.05 or <0.01 respectively of a one-tailed t-test compared with wild-type mice.

The ability to control the transgene expression made it possible, for the first time, to examine the role of ATF3 expression in adult mice hearts. Towards this end, we provided mice with doxycycline in the diet during mating thus suppressing ATF3 expression in the developing embryos. At weaning time (3 weeks of age), mice were either provided with regular water to express ATF3 (referred to as adult-ATF3 expressing mice) or left under a doxycycline-containing water as control (non-ATF3 expressing, double transgenic mice). As shown in Figure 2A, the control mice (non-ATF3 expressing) displayed a wild-type non-transgenic mice phenotype. Importantly, adult-ATF3 expressing mice displayed no apparent atrial phenotype (Figure 2B). However, they displayed a significant increase in their ventricle-to-body–weight ratio as soon as one week after doxycycline removal (Figure 2A), accompanied by an increased expression of the fetal gene program (Figure 2 C–F). While no significant difference in cell size is observed one week following doxycycline removal (not shown), cell size analysis displayed a significant increase in ventricular cell size following 6-20 weeks of ATF3 transgene expression (Figure 2 G-H). In addition, higher expression level of collagen type-Iα and TGFβ genes responsible for cardiac fibrosis was observed in the adult-ATF3 expressing mice (Figure 3 A-B). This is consistent with an increase in Masson’s trichrom staining, an indication for fibrosis (Figure 3 C-D). Echocardiography following up to four weeks of ATF3 expression displayed statistically significant and persistent reduction in heart function as evidenced by a smaller calculated FS: between 24% to 30% compared to ~ 40% in the control mice (Figure 3E).

**Figure 4:**
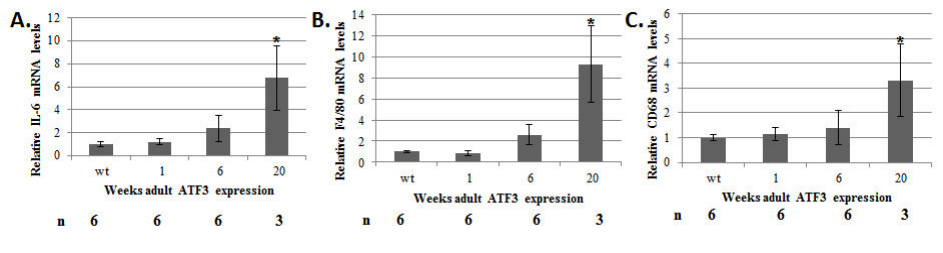
The hearts derived from adult-ATF3 expressing mice display a higher level of inflammatory response. mRNA derived from ventricles of wild-type and ATF3 transgenic, as described in Figure 2C, was analyzed for the indicated inflammatory markers **A**. IL-6 **B**. F4/80 C. CD68. The results represent the mean expression relative to GAPDH of the indicated number of animals (n). Asterisks (*) indicate a P value <0.05 of a one-tailed t-test compared with wild-type mice.

To examine the possibility of a cardiac inflammatory response in ATF3 expressing mice, we measured the level of inflammatory markers by RT-qPCR (Figure 4 A–C). IL-6 has previously been found to play a role in cardiac hypertrophy through the activation of the JAK-STAT pathway [21]. We found an increase in IL-6 production in the ATF3 transgenic mice following 6-20 weeks of exposure to ATF3 expression (Figure 4A). In addition, macrophage markers such as F4/80 and CD68 were highly elevated in the adult-ATF3 expressing mice at 20 weeks after doxycycline removal (Figure 4 B-C).

**Figure 5:**
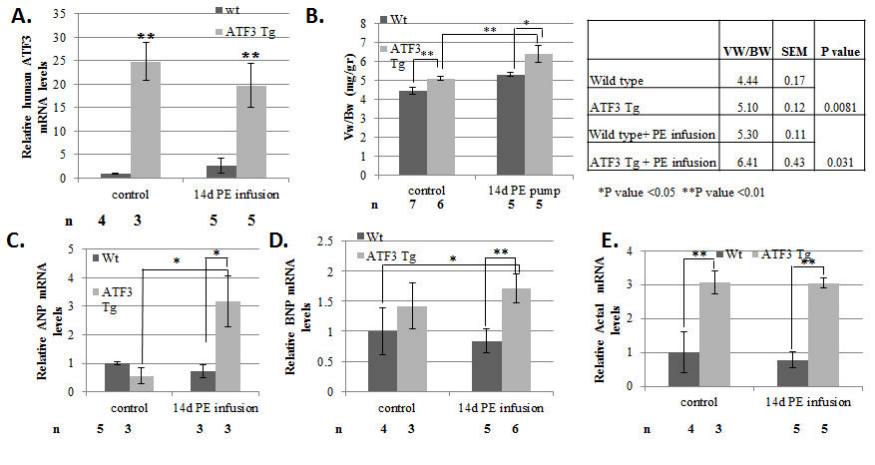
Adult-ATF3 expressing mice display increased Vw/Bw growth ratio in basal and following PE infusion. **A**. RT-qPCR analysis for cDNA derived from either wild-type or ATF3 transgenic mice. mRNA was extracted from ventricles derived from wild-type (black) or adult-ATF3 expressing (gray) and RT-qPCR was performed with the hATF3 specific primers. **B**. Mice ventricles weight (Vw) relative to mouse body weight (Bw) is calculated (mg/gr). The results represent the mean and SEM of the indicated number of animals (n). The mean and SEM of the absolute values is provided (right panel). **C**–**E**. RT-qPCR with cDNA from A was performed with the indicated specific primers: **C**. ANP. **D**. BNP. **E**. Skeletal actin (Acta1). The results represent the mean expression relative to GAPDH of the indicated number of animals (n). Asterisks (*/**) indicate a P value <0.05 or <0.01 respectively of a one-tailed t-test compared to wild-type mice.

**Figure 6:**
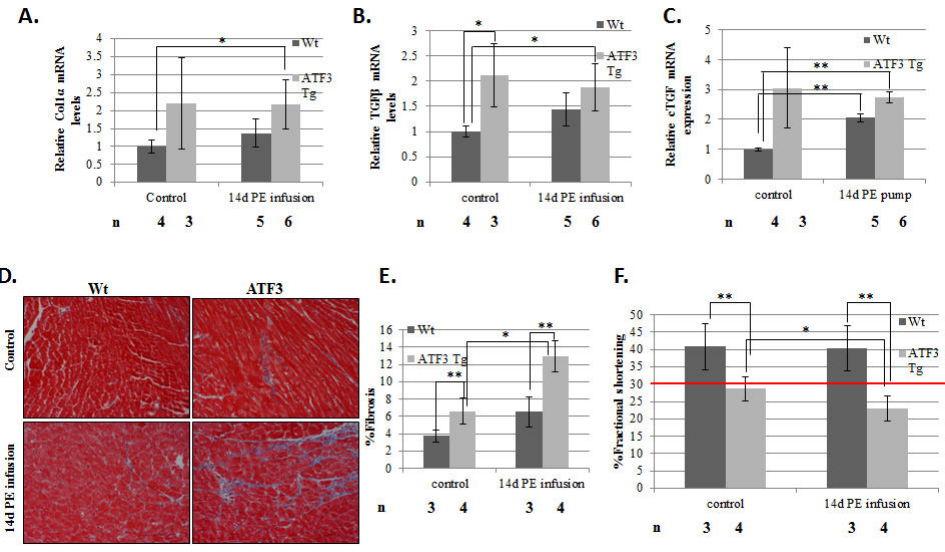
Adult-ATF3 expressing mice display higher fibrosis and lower heart function following a 2-week pressure overload model. RT-qPCR analysis for cDNA derived from either wild-type or ATF3 transgenic mice. mRNA was extracted from ventricles from wild-type (black) or ATF3 transgenic (gray) and RT-qPCR was performed with the indicated specific primers: **A**. Col1α **B**. TGFβ **C**. connective tissue growth factor (cTGF). The results represent the mean and SEM relative to GAPDH expression of the indicated number of animals (n). **D**. Masson trichrome staining of paraffin embedded sections of wild-type and adult-ATF3 expressing mice, either untreated (control) or after 2 weeks of PE infusion. **E**. Quantification of fibrosis of the indicated number of mice (n). At least five sections for the indicated number of mice (n) were analyzed **F**. Adult-ATF3 expressing mice treated as indicated were examined by micro-ultrasound and measurements were recorded to determine fractional shortening (FS) percentage in order to assess heart function. Maximal left ventricles end-diastolic (LVDd) and end-systolic (LVDs) dimensions parameters were measured in short-axis M-mode images. Fractional shortening (FS) was calculated as: FS (%) = [(LVDd-LVDs)/LVDd] X 100. The results represent the mean and SEM of the indicated number of animals (n). Asterisks (*/**) indicate a P value <0.05 or <0.01 respectively of a one-tailed t-test compared to wild-type mice.

We next examined the adult-ATF3 expressing mice under pressure overload insult by implanting Alzet mini-osmotic pumps that provide a constant two weeks infusion of phenylephrine (100 mg/kg/day). In the ATF3 transgenic mice, chronic PE infusion did not further increase the transgene ATF3 mRNA level, which was already high due to the transgene expression (Figure 5A). To test the combined effect ATF3 gain-of-function and chronic PE treatment, we first measured the heart-to-body–weight ratio. Both wild-type and ATF3-transgenic mice displayed significant increases in their ventricle-to-body-weight ratios upon PE treatment, interestingly to a similar extent, by 20% and 25%, respectively (Figure 5B). However, since the ventricle-to-body–weight ratio of ATF3-expressing mice was 20% higher at the basal level than the wild-type mice, the PE-treated ATF3-transgenic mice displayed a total of 45% increase in ventricle-to-body weight compared to untreated non-transgenic mice littermates. Significantly, PE infusion induced ANP fetal gene expression in the transgenic mice, but not in the control mice (Figure 5C). PE infusion did not induce the expression of the BNP and α-skeletal actin hypertrophic embryonic markers in the wild type mice nor the transgenic mice (beyond their already high basal level of these genes) (Figure 5 D-E). The pressure overload model resulted in an increase in fibrotic markers in wild-type mice, whereas no further increase in these markers was observed in adult-ATF3 expressing mice beyond their higher basal levels (Figure 6 A–C). Masson trichrom staining showed a significant increase in fibrosis in PE infused adult ATF3 expressing mice (Figure 6 D-E). Collectively, the ATF3 transgenic mice following PE infusion displayed a significant increase in heart mass and high levels of the ANP hypertrophic marker. This was also reflected in the deterioration of cardiac function: a drop of FS from approximately 30% to below 25% upon PE infusion for two weeks. This is in contrast to a stable heart function with 40% normal FS in wild type mice treated with two weeks of PE (Figure 6F).

**Figure 7:**
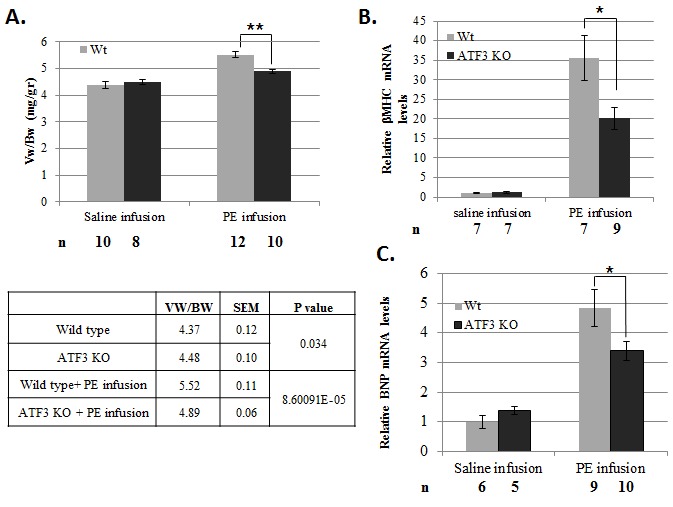
ATF3 KO mice display a lower rate of hypertrophy following two weeks of PE-induced pressure overload model. **A**. Mice ventricles weight (Vw) relative to mouse body weight (Bw) is calculated (mg/gr). The results represent the mean and SEM of the indicated number of animals (n) **B–C**. RT-qPCR analysis for cDNA derived from RNA extracted from ventricles of ATF3 KO and wild-type mice with the corresponding specific primers to indicated selected genes. **B**. βMHC. **C**. BNP. The results represent the mean and SEM expression relative to the GAPDH of the indicated number of animals (n). Asterisks (*/**) indicate a P value <0.05 or <0.01 respectively of a one-tailed t-test compared to wild-type mice.

To complement the above gain-of-function approach using transgenic mice, we used ATF3 KO mice [22] under the same pressure overload stress paradigm. Based on the results with adult-ATF3 expressing transgenic mice, we hypothesized that mice with a loss of ATF3 expression would display a reduced hypertrophic response. As shown in Figure 7A, in saline-infused mice, no significant difference was observed in the ventricle-to-body–weight ratio between ATF3 KO and wild-type mice. In addition, PE-infused wild-type mice exhibited a significant increase in the ventricle-to-body–weight ratio (by 26%). Importantly, ATF3 KO mice exhibited a significantly dampened hypertrophy response upon PE infusion (increase by 9%, Figure 7A). Consistent with the reduced cardiac hypertrophy in the ATF3 KO mice, the induction of hypertrophy markers βMHC and BNP in the ATF3 KO mice were significantly dampened in response to PE infusion, compared to the wild-type mice (Figure 7 B-C).

Collectively, our results show that ATF3 KO mice display reduced cardiac hypertrophy when challenged with chronic pressure overload induced by PE infusion. Taken together, our transgenic and KO mice data support a model that ATF3 promotes cardiac hypertrophy.

## Discussion

ATF3 is an immediate early transcription factor involved in cellular homeostasis through the regulation of genes encoding cellular response molecules and other transcription factors. ATF3 is induced in the heart in response to neuroendocrine hormones that result in an increase in blood pressure [11,13]. ATF3 is also induced in the heart of mice following ischemia/reperfusion [9]. In addition, in rat neonatal cardiomyocytes, ATF3 protects cradiomyocytes from doxorubucin-induced apoptosis [10]. However, the role of ATF3 in cardiac hypertrophy is controversial. The lack of ATF3 expression was shown to promote heart hypertrophy in response to the aortic banding pressure overload model [12], suggesting a role for ATF3 to suppress hypertrophy. Consistently, ATF3 was shown to play a role to suppress the expression of hypertrophic genes in isolated cardiomyocytes in a negative feedback loop [13]. In contrast, transgenic mice with ATF3 expression under the control of the αMHC promoter displayed atrial enlargement [9] and increased hypertrophic gene expression. Since ATF3 expression in these mice is initiated in the atria at embryonic day 10, it was not possible to distinguish between developmental defects and postnatal ATF3 activity. To better define the role of ATF3, we generated transgenic mice with an inducible cardiac expression of ATF3. This model allowed us to clearly distinguish between the phenotypes that occur during development versus those in adult upon transgene induction. In our embryonic-ATF3 expressing model, the newborn mice had enlarged atria, atrial fibrosis, hypertrophic markers expression, arrhythmia, and early death. This is consistent with the previous ATF3 transgenic mice [9].

The novel findings in the present study originate from the ability to suppress the ATF3 transgene expression during embryogenesis. The expression level of ATF3 in the postnatal ATF3-transgenic mice line is securely suppressed when mice are provided with doxycycline in their drinking water. This is apparent in newborn mice that were provided with doxycycline during embryogenesis, which displayed a normal cardiac phenotype. Upon doxycycline removal, the ATF3 mRNA level in the transgenic mice was highly expressed. In addition, mosaic staining of the ATF3 transgene expression was observed in cardiomyocytes by immunohistochemistry. This is a relatively common phenomenon in transgenic mice and is thought to be due to the position effect of the transgene [23]. Nevertheless, the ATF3 protein was barely detectable by Western blot analysis using either an anti-ATF3 or anti-HA antibodies. Therefore, the ATF3 transgenic mice represent a relatively modest exposure to chronic low ATF3 expression levels. In view of the central role of ATF3 in homeostasis maintenance [8], the ability to tightly and spatially control ATF3 expression in various tissues and organs would certainly be an extremely valuable research tool.

In just one week after doxycycline removal, the transgenic mice exhibited a higher ventricle-to-body–weight ratio, reaching a maximal 20% increase by 6 weeks following doxycycline removal. This was accompanied by the expression of hypertrophic markers that either remained high or moderately declined with time of exposure to ATF3 expression. In contrast, the expression of both fibrosis and inflammation gene markers significantly increased with time. Increased fibrosis and inflammatory response contributed to the maladaptive response to cardiac hypertrophy [24,25]. Interestingly, in previous studies, ATF3 was found to negatively regulate IL-6 in macrophages [26] and to mediate the repression of IL-6 transcription following heat-shock in mouse embryonic fibroblasts [27]. In contrast, ATF3 was found to upregulate IL-6 expression in pancreatic islets following hypoxia [28]. In the heart, we detected increased IL-6 mRNA levels with the time of exposure to adult-ATF3 expression. This increase in IL-6 transcript is correlated with the elevation of F4/80 and CD68 macrophage markers. However, we cannot exclude the possibility that the IL-6 transcript originates from the ATF3 expressing cardiomyocytes. Considering the role of macrophages in inflammatory response, the increase macrophage infiltration in the heart is likely to contribute to cardiac hypertrophy.

The exposure of adult ATF3 expressing mice to PE infusion (a pressure overload model) resulted in an additional increase in the ventricle-to-body–weight ratio and heart function deterioration along with the promotion of heart hypertrophy. Consistently, ATF3 KO mice exposed to pressure overload by PE infusion displayed significantly reduced hypertrophy compared to wild type mice. Taken together, our data support the model that ATF3 promotes cardiac hypertrophy. This is in contrast to the previous study by Zhou et al. which suggests that ATF3 suppresses cardiac hypertrophy [12]. This apparent discrepancy may be due to the differences between the pressure overload models used. Here, we used a mild two weeks pressure overload model compared with a robust four weeks aortic banding model. In the latter model, ATF3 protein level was clearly elevated at four weeks by about 4-5 fold [12]. In our model, however, we do not observe an increase in ATF3 mRNA or clear increase in the ATF3 protein level. This may explain the different phenotypes of ATF3 deficiency in the two pressure overload models. Although our transgenic mice are similar to the mice under aortic banding in that they both have clearly elevated ATF3 expression, they have different contexts. Aortic banding induces ATF3 expression but also other events that may not be present in the transgenic model. ATF3 was shown to be able to switch its function from a transcriptional repressor to an activator depending on its protein partner [5]. Therefore, it may be possible that in the aortic banding model, ATF3 cooperates with transcription factors that results in a switch in ATF3 function from a pro-hypertrophic to anti-hypertrophic activity. It would be of great interest identifying the ATF3 protein partners that facilitates its anti-hypertrophic activity in the aortic banding model. In addition, the aortic banding study by Zhou et al. used a loss-of-function approach to conclude anti-hypertrophic role of ATF3. However, here we demonstrated that from both gain- and loss-of-function approaches, ATF3 expression is consistent with its role in promoting cardiac hypertrophy in the PE infusion model. ATF3 is an adaptive response gene that maintains homeostasis and therefore can be beneficial in the short term. However, following sustained stress, ATF3 expression is detrimental and can contribute to heart disease development.

## Supporting Information

Figure S1 Embryonic-ATF3 expression results in enlarged atria phenotype. **A**. Mice genotyping by PCR was performed at 2 weeks of age. DNA was extracted from mice tails and a PCR reaction was performed with specific oligonucleotides to score the various genotypes. The number in parentheses represents the observed percentage for each genotype. χ^2^ tests were performed for all the possible genotypes based on the expected Mendelian distribution. A statistical difference was observed only for the embryonic ATF3-expressing group in which doxycycline was avoided during embryogenesis with a P value <0.05. **B**. Survival curve for ATF3 transgenic mice (n=12) in which ATF3 was expressed during embryonic development were followed up to 60 days. **C**. Hearts from newborn mice untreated with doxycycline were harvested and photographed at 4 weeks of age. **D**. Atria and ventricles derived from either wild-type (black) or ATF3 transgenic mice (gray) were separated and weighted. The ventricles weight (Vw) and atrial weight (Aw) relative to body weight (Bw) were calculated (mg/gr). The results represent the mean and SEM of the indicated number of animals (n). ** P value <0.01 of a one-tailed t-test compared with wild-type mice. **E**. Electrocardiograph (ECG) recordings of either a wild-type mouse (upper panel) or an embryonic ATF3 expressing mouse (lower panel). The arrow shows the loss of normal P-wave that indicates an arrhythmia.
(TIF)

Figure S2 Embryonic-ATF3-expressing survivor mice display high levels of hypertrophic markers and increased cell size. **A**. RT-qPCR analysis for cDNA derived from either wild-type or embryonic ATF3 expressing mice. Mice were sacrificed at 60 days after birth and mRNA was extracted from either atria or ventricles (Vent). RT-qPCR was performed with brain natriuretic peptide (BNP) specific primers. The results represent the mean and SEM from the indicated number of animals (n). **B**. Heart sections were stained with TRITC-labeled wheat-germ agglutinin. Representative sections are shown. **C**. Cell size was analyzed using Image Pro Plus software. At least five areas per section were analyzed for the indicated number of mice (n). **D–F** RT-qPCR with specific oligonucleotide corresponding to: **D**. Transforming growth factor β (TGFβ) **E**. collagen1 α (col1α) **F**. Connective tissue growth factor (cTGF). The results represent the mean and SEM of the indicated n number of animals (n). Asterisks (*/**) indicates P values <0.05 or <0.01 respectively of a one-tailed t-test compared with wild-type mice.
(TIF)

Figure S3 Embryonic ATF3 expressing survivor mice display lower levels of MLC2a, connexin 40 and heart function A. RT-qPCR from mRNA from atria, as described in Figure S2, with specific oligonucleotide corresponding to: **A**. Atrial myosin light chain (Mlc2A) **B**. Connexin 40. **C**. Embryonic ATF3 expressing mice were examined by micro-ultrasound and measurements were recorded to determine fractional shortening (FS) percentage in order to assess heart function. Maximal left ventricles end-diastolic (LVDd) and end-systolic (LVDs) dimensions parameters were measured in short axis M-mode images. Fractional shortening (FS) was calculated as: FS (%) = [(LVDd-LVDs)/LVDd] X 100. Echocradiography measurements were performed at three weeks of age. The results represent the mean and SEM of the indicated number of animals (n). Asterisks (**) indicates P values <0.01 of a one-tailed t-test compared with wild-type mice.
(TIF)
