# Correction: Adult Cardiac Expression of the Activating Transcription Factor 3, ATF3, Promotes Ventricular Hypertrophy

**DOI:** 10.1371/annotation/87e2a80b-3ed7-4ef9-96cb-1268d91b6366

**Published:** 2013-09-19

**Authors:** Lilach Koren, Ofer Elhanani, Izhak Kehat, Tsonwin Hai, Ami Aronheim

A Funding Statement was incorrectly omitted from the article. The Funding Statement is: "This work was supported by grant No. MH-3-7224 from the Chief Scientist’s office of the Ministry of Health, Israel to AA and by Grant No. 2009179 from the United States-Israel Bi-national Science Foundation (BSF) to TH and AA. We are also thankful for the support from the Jees & Midred Fisher Family’s Cardiology Research Fund. The funders had no role in study design, data collection and analysis, decision to publish, or preparation of the manuscript." 

