# Supplementary figures and images for: Adult Cardiac Expression of the Activating Transcription Factor 3, ATF3, Promotes Ventricular Hypertrophy
Source: PLoS One. 2013 Jul 3;8(7):e68396. doi: 10.1371/journal.pone.0068396 (PMC3707568; doi:10.1371/journal.pone.0068396)

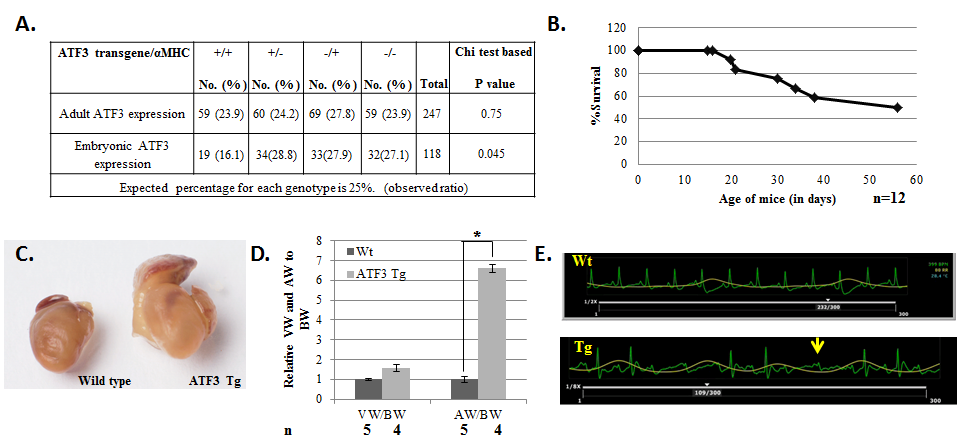

Supplement: Figure S1 — A. Mice genotyping by PCR was performed at 2 weeks of age. DNA was extracted from mice tails and a PCR reaction was performed with specific oligonucleotides to score the various genotypes. The number in parentheses represents the observed percentage for each genotype. χ2 tests were performed for all the possible genotypes based on the expected Mendelian distribution. A statistical difference was observed only for the embryonic ATF3-expressing group in which doxycycline was avoided during embryogenesis with a P value <0.05. B. Survival curve for ATF3 transgenic mice (n=12) in which ATF3 was expressed during embryonic development were followed up to 60 days. C. Hearts from newborn mice untreated with doxycycline were harvested and photographed at 4 weeks of age. D. Atria and ventricles derived from either wild-type (black) or ATF3 transgenic mice (gray) were separated and weighted. The ventricles weight (Vw) and atrial weight (Aw) relative to body weight (Bw) were calculated (mg/gr). The results represent the mean and SEM of the indicated number of animals (n). ** P value <0.01 of a one-tailed t-test compared with wild-type mice. E. Electrocardiograph (ECG) recordings of either a wild-type mouse (upper panel) or an embryonic ATF3 expressing mouse (lower panel). The arrow shows the loss of normal P-wave that indicates an arrhythmia. (TIF) [file pone.0068396.s001.tif]

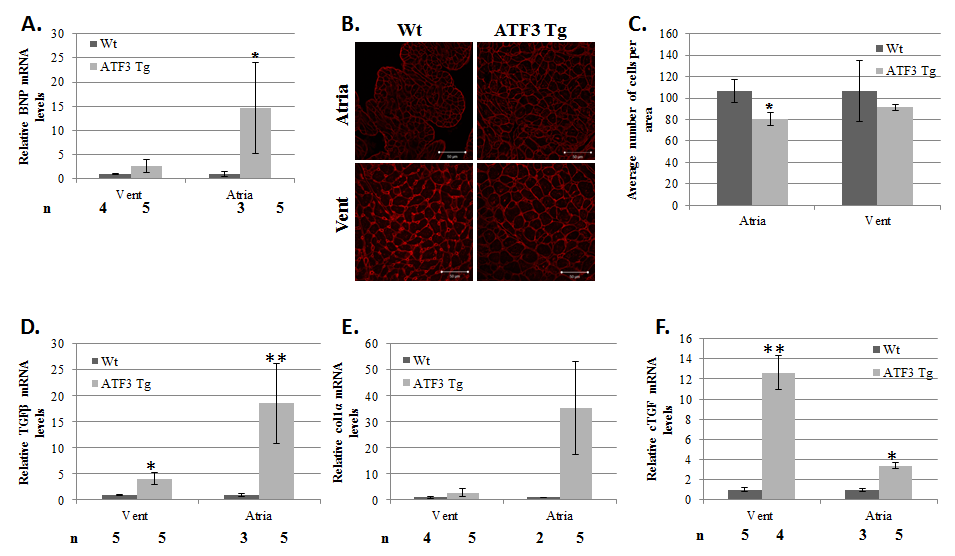

Supplement: Figure S2 — A. RT-qPCR analysis for cDNA derived from either wild-type or embryonic ATF3 expressing mice. Mice were sacrificed at 60 days after birth and mRNA was extracted from either atria or ventricles (Vent). RT-qPCR was performed with brain natriuretic peptide (BNP) specific primers. The results represent the mean and SEM from the indicated number of animals (n). B. Heart sections were stained with TRITC-labeled wheat-germ agglutinin. Representative sections are shown. C. Cell size was analyzed using Image Pro Plus software. At least five areas per section were analyzed for the indicated number of mice (n). D–F RT-qPCR with specific oligonucleotide corresponding to: D. Transforming growth factor β (TGFβ) E. collagen1 α (col1α) F. Connective tissue growth factor (cTGF). The results represent the mean and SEM of the indicated n number of animals (n). Asterisks (*/**) indicates P values <0.05 or <0.01 respectively of a one-tailed t-test compared with wild-type mice. (TIF) [file pone.0068396.s002.tif]

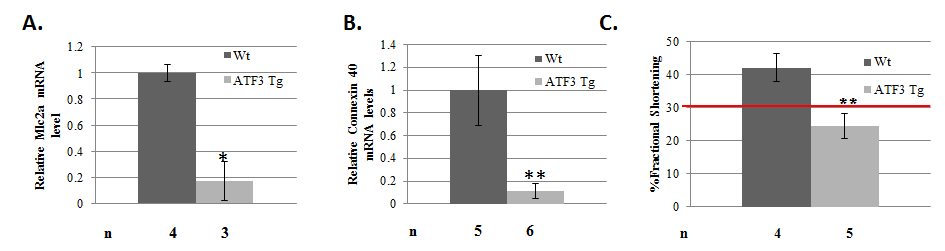

Supplement: Figure S3 — RT-qPCR from mRNA from atria, as described in Figure S2, with specific oligonucleotide corresponding to: A. Atrial myosin light chain (Mlc2A) B. Connexin 40. C. Embryonic ATF3 expressing mice were examined by micro-ultrasound and measurements were recorded to determine fractional shortening (FS) percentage in order to assess heart function. Maximal left ventricles end-diastolic (LVDd) and end-systolic (LVDs) dimensions parameters were measured in short axis M-mode images. Fractional shortening (FS) was calculated as: FS (%) = [(LVDd-LVDs)/LVDd] X 100. Echocradiography measurements were performed at three weeks of age. The results represent the mean and SEM of the indicated number of animals (n). Asterisks (**) indicates P values <0.01 of a one-tailed t-test compared with wild-type mice. (TIF) [file pone.0068396.s003.tif]
